# Sclerosing peritonitis associated with luteinized thecoma and elevated serum CA 125 levels: case report

**DOI:** 10.1590/S1516-31802008000200011

**Published:** 2008-03-06

**Authors:** Sabas Carlos Vieira, Lina Gomes dos Santos, Felipe José da Silva Melo Cruz, Wildson Moura Gonçalves, Sheila de Farias Viana Castelo Branco Rocha

**Keywords:** Thecoma, Peritonitis, Ovarian neoplasms, CA-125 antigen, Germinoma, Tecoma, Peritonite, Neoplasias ovarianas, Antígeno Ca-125, Germinoma

## Abstract

**CONTEXT:**

Thecomas are benign tumors that account for less than 1% of all ovarian neoplasms. The association of ovarian thecoma with sclerosing peritonitis is rare.

**CASE REPORT:**

We report the case of a 33-year-old woman, with a two-month history of increasing abdominal volume. Ultrasound showed a complex pelvic lesion and laboratory analysis detected elevated serum CA 125 levels. The patient underwent total abdominal hysterectomy with bilateral salpingo-oophorectomy and peritoneal biopsy. Histopathological analysis revealed the presence of luteinized thecoma of both ovaries associated with sclerosing peritonitis.

## RESUMO

**CONTEXTO:**

Os tecomas são tumores benignos e representam menos de 1% das neoplasias ovarianas. A associação de tecoma de ovário com peritonite esclerosante é rara e caracteriza-se pela presença de múltiplos espessamentos nodulares fibróticos no peritôneo.

**RELATO DE CASO:**

Relatamos um caso de uma paciente de 33 anos de idade, com aumento de volume abdominal há dois meses. A ultra-sonografia mostrou uma lesão pélvica complexa e a dosagem de CA 125 mostrava-se elevada. A paciente submeteu-se a histerectomia abdominal total com ooforectomia bilateral e biópsia peritoneal. O exame histopatológico revelou a presença de tecoma luteinizado de ovário bilateral associado a peritonite esclerosante.

## INTRODUCTION

Thecomas are rare neoplasms that usually occur in postmenopausal women. Luteinized thecoma of the ovary is characterized by a group of polygonal cells with clear, vacuolated and abundant lipid-filled cytoplasm linked to tumor estrogen activity.^1^ This neoplasm may be associated with multiple fibrotic nodular thickening of the peritoneum, which is named sclerosing peritonitis. Association of these two conditions is extremely rare and the etiology currently remains unclear. Patients may present with abdominal distension, intestinal obstruction, palpable unilateral or bilateral mass, weight loss, nausea and vomiting.^2^

The purpose of the present paper is to report on a rare association between thecoma of the ovary and sclerosing peritonitis with increased serum CA 125 levels.

## CASE REPORT

A 33-year-old woman who had had five pregnancies, three deliveries and two abortions, sought medical treatment for a two-month history of increasing abdominal volume and weight loss of 4 kg in a month. On physical examination, a palpable mass was noted in the left iliac fossa with clinically apparent tumor infiltration in the umbilical scar. Ultrasound revealed a complex pelvic lesion. Measurement of serum CA 125 detected elevated levels (316.9 U/ml). The patient underwent exploratory laparotomy, which showed an ovarian tumor with multiple implants in the peritoneal cavity, suggesting that it was malignant. There was also thickening of the peritoneum in the umbilical scar region.

Intraoperative biopsy showed that the tumor was benign. However, based on the clinical and surgical findings, as well as the elevated serum CA 125 levels, we chose to perform total abdominal hysterectomy with bilateral salpingo-oophorectomy, peritoneal biopsy and removal of a fragment from the greater omentum and a nodule from the abdominal cavity.

Macroscopically, the outer surface of the right ovary appeared lobulated and grayish-white with granular areas, measuring 15 cm across its largest diameter. The sectioned surfaces of the right ovarian mass were solid, yellow and granular with mucin-containing cystic areas. The outer surface of the left ovary appeared undulating and was grayish-white, measuring 3.8 cm in its largest diameter. Sections through the left ovarian mass revealed a fibrillar and grayish-white surface.

**Figure 1:**
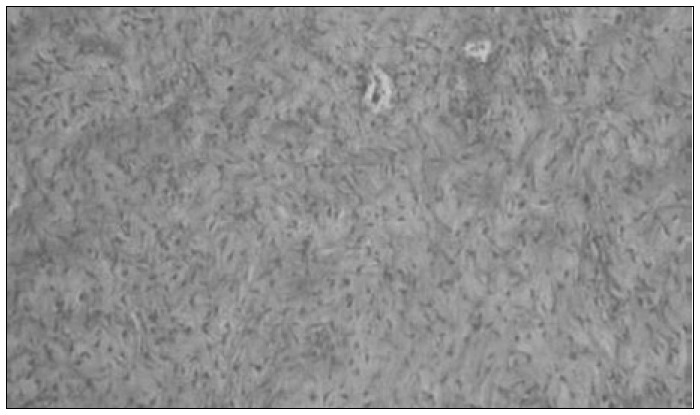
Photomicrograph showing luteinized thecoma of the ovary (hematoxylin-eosin; 100 x).

**Figure 2:**
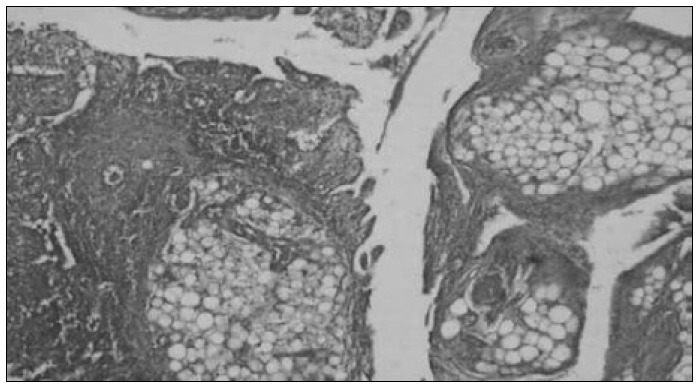
Photomicrograph showing peritoneum with intense fibrosis (hematoxylin-eosin; 40 x).

Histological examination of the ovaries identified a benign tumor composed of rich fibromatous stroma with rare isolated lutein cells and low mitotic index (Figure 1). The peritoneum and greater omentum showed extensive fibrous connective tissue proliferation associated with mononuclear inflammatory infiltrate (Figure 2), thus establishing the diagnosis of sclerosing peritonitis.

The patient remains asymptomatic 18 months after surgical treatment.

## DISCUSSION

Luteinized thecomas are ovarian tumors composed of polygonal cell proliferation with clear vacuolated cytoplasm, filled with abundant lipid droplets that are linked to tumor estrogen activity.^1^ In general, they occur in younger women (30% in women under 30 years of age) than do typical thecomas.^2^ Thecomas are virtually always unilateral and are linked to estrogen or androgen activity in 61% of the cases.^3^

Luteinized thecomas are tumors that mostly exhibit benign behavior. In rare cases, they may be associated with sclerosing peritonitis. Such an association has been described in a total of 18 cases in the literature.^1^ The process of peritonitis consists of fibroblastic and myofibroblastic cell proliferation separated by collagen, fibrin and occasionally mononuclear inflammatory cells.^1^ The role of hormones in the etiology of sclerosing peritonitis is yet to be defined, since many tumors with hormonal activity, such as granulosa cell tumors, are not accompanied by sclerosing peritonitis. It is possible that luteinized thecomas secrete an undetermined substance that stimulates fibroblast proliferation in the peritoneum.^2^ The two most commonly known causes of sclerosing peritonitis are chronic ambulatory peritoneal dialysis, possibly due to chlorhexidine in the dialysate, and practolol therapy.^3^ In the present report, the etiology was not identified.

The clinical manifestations may be due to either ovarian thecoma or peritonitis, or both. The symptoms include ascites, abdominal distension, intestinal obstruction, abdominal pain, palpable mass and weight loss.^1^ In the present case, all these symptoms were observed, except intestinal obstruction and ascites. The laboratory analysis showed the presence of elevated serum levels of CA 125 (316.9 U/ml), which is unusual in benign neoplasms such as luteinized thecomas. To date, only one other case of luteinized thecoma with sclerosing peritonitis associated with increased levels of CA 125 has been reported in the literature.^3^

The differential diagnosis for sclerosing peritonitis includes a group of diseases presenting as tumor masses that are confined to the mesentery. Peritoneal metastases are among these disorders. Differentiating this condition from other diseases is of vital importance, since the therapeutic approach and prognosis vary greatly.^2^

Sclerosing peritonitis is the major factor contributing towards the morbidity and mortality of patients with luteinized thecoma, since multiple surgical procedures are required in order to manage the bowel obstruction that these adhesions cause.^1^ Our patient had a good postoperative course and no further surgical interventions have been needed to date.

The treatment for this disease has still not been established because it is a rare condition that has been the subject of very few studies. In the literature, there are no cases describing the influence of hormones on the development of this type of tumor. However, it is possible that circulating estrogens may play a role in expressing sex steroid receptors in these tumors.^1^ The role of some drugs like tamoxifen and hydrocortisone has been evaluated.^4,5^

## CONCLUSION

We reported a case of luteinized thecoma with sclerosing peritonitis, which is a rare benign pathological condition. Because of the presence of multiple fibrotic nodular thickening of the peritoneum and, in some cases, high serum CA 125 levels, it is of fundamental importance to differentiate it from peritoneal metastases.
